# A Practical Guide to the Study of the Diseases of the Eye: Their Medical and Surgical Treatment

**Published:** 1862-07

**Authors:** 


A Practical Guide to the Study of the Diseases of the Eye: Their Medical and Surgical Treatment. By Henry W. Williams, M.D., Fellow of the Massachussets Med. Society, Honorary Fellow of the Rhode Island Med. Society, Member of the American Med. Association, etc., etc. Boston: Tichnor & Fields. 1862.

This is a small sized octavo volume of 317 pages. The publishers have done their part of the work in excellent style; and though we have not had time to examine the contents of the work carefully, we have no doubt but it is a very valuable practical treatise. And from its conciseness, it will be found one of the most convenient, as well as reliable, works that the practitioner can obtain.

The following paragraph from the preface will convey a good idea of the work:—

"It has been the aim of the author to avoid encumbering his work, and confusing the reader, by the introduction of merely exceptional details,—by an account of every proposed but exploded mode of treatment, or by more than the most sparing use of the too learned technical designations which abound in ophthalmic literature; but he has endeavored to give clear and explicit descriptions of the usual forms of disease, so that the physician my be able to recognize, at once, their distinctive features,—and to define the course of treatment best adapted, in a majority of cases, to remove the morbid condition. Some affections, and certain phase of disease, of rare occurrence and trivial importance, will be merely alluded to; enough being said, however, to enable the reader to detect their character, as exceptional cases, and to allow him to consult other works, at his leisure, should he wish for complete information regarding them."

The book is for sale by S. C. Griggs & Co., of this city.

